# Spinal pain prevalence in Wales and associated risk factors.

**DOI:** 10.23889/ijpds.v7i3.1900

**Published:** 2022-08-25

**Authors:** Hywel Turner Evans, Ian Farr, David Byfield, Benjamin Stacey, Damian Bailey

**Affiliations:** 1 SAIL Databank, Swansea University Medical School, Swansea, Wales; 2 Neurovascular Research Laboratory, Faculty of Life Sciences and Education, University of South Wales, Pontypridd, Wales

## Objectives

Spinal pain predisposes patients to a more sedentary lifestyle, a major risk factor for cardiovascular disease and other comorbidities. There is little recent evidence of the current prevalence of spinal pain and associated risk factors in Wales. This analysis addresses this gap in knowledge.

## Approach

This retrospective e-cohort study used linked National Survey for Wales (NSW) data and Welsh Demographic Services data held in the Secure Anonymised Information Linkage (SAIL) Databank. All years of the annual NSW data from 2016 to 2020 (N = 34,123) were used to determine the prevalence of spinal pain in Wales. The likelihood of developing spinal pain was quantified by multivariate regression cross-sectional analysis, adjusting for the presence of the same person in multiple years of the survey data. Predictors included socio-demographic and health status, including mental health and cardiovascular disease.

## Results

Spinal pain affected 5% of people who took part in the NSW. This analysis also shows that spinal pain disproportionally affects some sub-populations of Wales. Factors associated with a greater likelihood of spinal pain were cardiovascular disease, presence of at least one mental health condition, living in a more deprived area, and education level. This is especially pertinent as the burden of cardiovascular risk is disproportionately elevated in the Welsh population and Wales represents a distinctive demographic, characterised by geographical constraints and low socio-economic status. These factors will be presented and discussed in detail.

## Conclusion

The prevalence of spinal pain and associated risk factors in Wales was quantified. This work will help inform public health action to encourage interventional and prevention strategies to improve the quality of life for those suffering with spinal pain across Wales.

